# Judgment

**Published:** 1897-06

**Authors:** C. B. Blackmarr

**Affiliations:** Jackson, Mich.


### Judgment.

C. B. BLACKMARR, D.D.S., JACKSON, MICH.

Read before the Southwestern Dental Society of Michigan, April 14, 1897.

Gentlemen of the Dental Association of Southwestern Michigan:—When I received the invitation from your Secretary, to write something for this meeting, I was arguing with my operative assistant, that to be successful, more judgment was required in dentistry to determine what to do, than to perform the operation. And in this discussion, cases that we remembered in our practice, and others, were brought up, which showed that skill, time and money were wasted or sacrificed just because more judgment was not used before beginning the operations, so much so, that I then thought "Judgment" good or bad in dentistry, would be a good subject for me to present to you to-day.

I took the dictionery and looked up the meaning of the word judgment. "It is the mental faculty by which man ascertains the truth by comparing facts and ideas." Now, I say that this particular mental faculty should be used more in connection with our skill and work. Prof. McGraw, of Detroit, said to us at the tri-state meeting there, that there seemed to be plenty of work for the dentists to do, and that all that seemed necessary for the dentists to do to be successful, was to learn how to treat teeth without pain, and to do it cheap.

Now, in my mind that is good judgment. We all want to be successful. Why is it that we cannot do both? Well, it is mostly because we do not use good judgment. We are obliged to use good gold and material generally. Why not put more good judgment in with our other good material?

And now, I want to call your attention to the fact that your judgment can be strengthened in any case you have in hand, by getting advice from the dental faculty at our University of Michigan, or any other college where they teach such matters. They are as willing to give dentists the benefit of their experience, observation and advice, as the Michigan Agricultural College does to farmers, and I believe with just as much benefit.

Now, why don't we treat teeth without pain? Because good judgment is lacking in patient or operator, and sometimes in both. I say patient as well as operator, because there are patients who will not allow the operator to make a thorough operation. They would not feel natural if anything was done well around them. They are bound to have a big, bad time. In a sense they seem to enjoy it. These patients need to be educated. Dentistry is either well or ill done. And it depends so much upon the class of patients a dentist has which it is to him. Good judgment is not used by the operator when he makes tedious, careless and painful operations. Also when he does not keep his appointments sharply. Also when he leaves his patient tied up with rubber dam to attend to other matters. Also wasting his time when waiting for water to heat, which he wishes to use; putting alcohol in his lamp; rolling his gold; mixing his amalgam, cement, etc., during the hour of operation, when an assistant could have them all ready at the instant needed. I tell you gentlemen, that one of the most painful things a patient has to endure, is to wait for the operator to perform his work. So much time is often wasted in trying to adjust the rubber dam, that the patient is tired out before the cavity in the tooth is touched. When, with an assistant and proper clamps, it could have been done in an instant.

Another thing I wish to say, and that is, skill, judgment, instruments and appointments, in general, which enable the operator to perform the operation with dispatch, will do more to relieve pain than any other way known to dentists "up to date."

A dentist *often* feels poor (I have the word "often" underscored) and imagines that his patient feels the same way, and so gives him cheap work, just because he is afraid the patient will not be willing to pay for the best he could do. This is again poor judgment and management. Also, "it is not so much how much he pays, as what he gets for what he pays." This makes me think of Bill Nye's grocery where he had this sign out, "Eggs 8 cents. Good Eggs 15 cents." And I think if a dentist had a similar sign out in regard to his operations, patients generally would make as good a choice in quality of work to have done, as they would in the quality of eggs to be bought.

And why don't we make many of our operations cheaper, as Prof. McGraw suggests? Principally because we waste so much time doing them. We seem to learn and have judgment and knowledge in two ways, observation and experience. Observation seems to be the cheapest teacher. I have been fortunate or unfortunate in always having assistants to help me in my work at dentistry, and I have seen so many times that my operative assistants would waste their time and skill from lack of using judgment, and not studying the cases in regard to the results of work done. How many times we have seen teeth treated for months, that should have been extracted at once. I once remember one of my assistants requesting a young man to come each day for two weeks and have a certain tooth medicated. This was in mid-winter, and I noticed the patient looked so cold each day, and I chanced to ask him how far he lived out of the city, and he said eighteen miles. Now, that was poor judgment on my operator's part, because the tooth could have been saved with less expense to the patient. How many times we see an operator trying to perform some operation, and making a hard, big matter of it, when a little consideration would have simplified the whole affair.

Did you ever see an operator trying to see into a cavity by swabbing out with cotton, dozens and dozens of times, when a rubber dam would have kept it dry at once? Did you ever see teeth treated for months carelessly, when one or two good, thorough treatments would have ended it all? How many times have you seen teeth filled that should have been extracted for the good of other teeth, and again extracted when they ought to have been saved, because needed to antagonize other teeth. We see so many times teeth extracted that should be regulated, plates made when bridges ought to be and visa versa, all gold fillings when tin and gold would be better, all for want of using judgment with the material used.

And children's teeth—how often have we seen temporary teeth pulled out long before they were ready to come out, making future trouble for the patient.

I think, gentlemen, that it requires more judgment to tell when to pull a tooth, than how to pull it. So I say that we as dentists, should consider the future results of our work to our patients, if we would wish to have our reputation such that our patients think, that for the time and money spent by them, they have had good results.

A prominent discussion was once being had in regard to root filling material. Finally an old practitioner ended the discussion by saying as you may remember, "Gentlemen, the best material for filling root canals is judgment."

